# Clinical symptoms and molecular epidemiologic characteristics of varicella patients among children and adults in Ganzhou, China

**DOI:** 10.1186/s12985-025-02661-6

**Published:** 2025-02-21

**Authors:** Ting Zeng, Chao-Xian Lian, Xiao-Yi Zhang, Ping-Qing Liu, Jian Ao, Gang-Feng Zhou, Xiao-Dong Chen, Dan-Dan Huang, Dian-Gui Hu, Xin Chen

**Affiliations:** 1 Department of Pathogenic Biology, School of Basic Medical Sciences, Gannan Medical University, Ganzhou, 341000 China; 2 Department of Medical Laboratory, Qianxi People’s Hospital, Qianxi, 551500 China; 3 Department of Epidemiology, School of Public Health and Health Management, Gannan Medical University, Ganzhou, 341000 China; 4 Department of Infectious Disease, Ganzhou Fifth People’s Hospital, Ganzhou, 341000 China

**Keywords:** Age group, Complication, Ganzhou, Genotype, Varicella-Zoster virus

## Abstract

**Background:**

Varicella-zoster virus (VZV) is highly transmissible; however, there are limited studies in China.

**Methods:**

The clinical symptoms, disease progression, and laboratory test results of varicella patients among children and adults diagnosed at Ganzhou Fifth People’s Hospital from August 2021 to December 2022 were analysed retrospectively. Genetic polymorphisms in the open reading frame (ORF) 22 and ORF62 fragments of VZV isolates were analysed using molecular epidemiological methods.

**Results:**

Thirty-nine varicella patients were included in this study, 26 of them were children and 13 of them were adults. The incidence of discomfort and complications was significantly greater in adults than in children (*P* < 0.05). Four adults developed severe disease, one of whom died, with no cases of severe disease or death among children. The 32 VZV clinical isolates were all Clade 2 wild-type strains. Four variant isolates from children had eight base mutations, five of which were missense; two variant isolates from adults had four base mutations, all of which were missense.

**Conclusions:**

The risk of developing severe disease or even death after VZV infection in adults was greater than that in children. There is an urgent need for more studies focusing on the differences in the pathogenicity of VZV in different age groups.

**Supplementary Information:**

The online version contains supplementary material available at 10.1186/s12985-025-02661-6.

## Background

Varicella-zoster virus (VZV) is a highly transmissible double-stranded linear DNA virus, approximately 125 kb in length, belonging to the Alphaherpesvirinae subfamily of the Orthoherpesviridae family. Upon initial infection of the human body, VZV causes viremia and spreads throughout the body, leading to chickenpox, which is characterized by symptoms such as fever, headache, and a distinctive rash [1]. Subsequently, the virus spreads through the bloodstream to sensory ganglia, such as the dorsal root ganglia and trigeminal ganglia, entering a long-term latent state where it ceases replication and causes no symptoms [2]. When the body’s immune system is weakened, the virus can reactivate, migrating along sensory nerves to the skin and causing shingles [3]. The symptoms of shingles include severe pain and a vesicular rash that follows the distribution of the affected nerves. Additionally, postherpetic neuralgia may occur as a major complication [4]. Although most cases of varicella are relatively mild at presentation, they can become severe or fatal by causing serious complications, such as pneumonia and encephalitis [5]. Several studies have shown that the severity of disease in VZV-infected individuals tends to correlate with age at the time of infection, with younger and older people generally being more susceptible and at greater risk of severe disease. Immunocompromised or deficient individuals, such as HIV-infected individuals, are also at high risk of developing severe disease or even dying after infection [6–8]. There are significant differences, with primary VZV infection usually occurring in childhood in temperate regions, whereas in tropical regions, the first infection is delayed until adulthood [9]. Differences in immune function and response mechanisms to the virus between children and adults may lead to differences in disease symptoms, disease progression, genotypes of infection, and treatment of primary VZV infection.

Since the 1950s, researchers began isolating and characterizing VZV. In 1986, Davison and Scott successfully deciphered the complete nucleotide sequence of VZV (Dumas strain) [10, 11]. Subsequently, VZV was found to have different genotypes, and according to the new nomenclature of VZV proposed by Breuer et al. in 2010, VZV can be categorized into five established evolutionary Clades (Clades 1–5) and two provisional evolutionary Clades (Clades VI and VII) [12]. There were certain genetic variations and geographic distribution differences among these Clades: Clades 1 and 3 were the predominant evolutionary branches in Europe and the Americas; Clades 4 and 5 were found mainly in the tropical regions of Africa and Central America; Clade VI was found in France and Italy located in southern Europe; and Clade VII was isolated only in the United States [13–16]. Many studies have shown that VZV Clade 2 was overwhelmingly prevalent in Asia, and a live attenuated vaccine used to prevent infections in China was developed on the basis of the Oka strain of Clade 2 [17–20].

Genotyping of VZV could help reveal the genetic diversity, evolutionary patterns, and transmission pathways of the virus [21–23]. Since genetic variation among different viral strains of VZV consists of almost unevenly distributed single-nucleotide changes, rapid genotyping of isolates could be performed by single-nucleotide polymorphism (SNP) mapping of highly variable regions of the VZV genome [22–26]. SNP sites based on a short fragment (447 bp) of the open reading frame (ORF) 22 had been reported to distinguish three major genotypes of VZV: E (European, Clade 1 or 3), J (Japanese, Clade 2), and M (Mosaic, Clade 4 or 5) [23]. Furthermore, a comparison of the differences in gene sequences between the Oka vaccine strain and the parental Oka strain revealed that over one-third of the nucleotide differences were in ORF62 [22, 23]. Therefore, SNP analysis of the ORF62 fragment could distinguish the parental Oka strain from the vaccine strain, both of which belonged to Clade 2.

Located inland approximately 400 km north of Hong Kong, China, Ganzhou is the largest city in Jiangxi Province, with a population of nearly 9 million. In this study, the clinical symptoms, disease progression, and laboratory test results of varicella patients among children and adults in Ganzhou from August 2021 to December 2022 were retrospectively analysed, and the genetic diversity of the isolates was characterized. The disease differences between different age groups infected with VZV were investigated, and the molecular epidemiological characteristics of VZV were clarified to provide a reference for prevention and treatment.

## Materials & methods

### Study population and sample collection

Varicella patients, including those with varicella primary cases and varicella breakthrough cases, were diagnosed at the Department of Infection of Ganzhou Fifth People’s Hospital from August 2021 to December 2022. Information on all patients’ medical records, including age, sex, infection time, patient source, clinical symptoms, complications, and laboratory test results, was collected. Whole blood was collected from patients, and the plasma was centrifuged and stored at -80 °C until subsequent experiments.

### Amplification of VZV fragments

Since viremia usually occurs during varicella, viral DNA was directly extracted from the plasma samples using the TaKaRa MiniBEST Viral RNA/DNA Extraction Kit Version 5.0 (TaKaRa, Japan). None of the samples had undergone virus isolation. The VZV ORF22 and ORF62 fragments were amplified by nested polymerase chain reaction using TransTaq^®^ DNA Polymerase High Fidelity (TransGen, China); the amplification primers were shown in Table 1 [27, 28]. For the ORF22 fragment, the first amplification conditions were as follows: 96 °C for 10 min; 33 cycles of 95 °C for 30 s, 58 °C for 30 s, and 72 °C for 40 s; and 72 °C for 10 min. For the second amplification conditions, the cycling conditions were the same, except that the annealing temperature was 60 °C and the incubation at 72 °C in each cycle was 50 s. For the ORF62 fragment, the first amplification conditions were as follows: 96 °C for 10 min; 34 cycles of 95 °C for 30 s, 55 °C for 30 s, and 72 °C for 2.5 min; and 72 °C for 10 min. For the second amplification conditions, the cycling conditions were the same, except that the annealing temperature was 60 °C, and the incubation at 72 °C in each cycle was 2 min. The amplified products were tested with a 1% agarose gel. The possible positive samples were purified and sequenced by Tsing Ke Biotech Co., Ltd. (Kunming, China).

**Table 1:** Primers used for nested PCR in ORF22 and ORF62

ORF	Primer name	Sequence(5´→3´)	Position ^*^	Amplicon (bp)
22	ORF22-W1F	AACAAACGACTCGGGTTTTG	37,825–37,844	652
	ORF22-W1R	AGTTTAGCCGTTTCGTGTGC	38,457–38,476	
22	ORF22F	TAGCATGTCTGGAGGCAATGG	37,870–37,890	359
	ORF22R	ATCGGCGGATAATAACACAACC	38,207–38,228	
62	ORF62-W1F	ATTACTCTGGGAATCGGGGG	105,607–105,626	1908
	ORF62-W1R	TATAGCATGGCTCCAGAACCC	107,494–107,514	
62	ORF62F	GGCCACATTACTCTGGGAAT	105,601–105,620	1793
	ORF62R	GTTCCGCATGTAGGCGAAG	107,375–107,393	

^*^ According to the reference sequence of Dumas (X04370)

### Phylogenetic analyses of VZV sequences

The sequences of ORF22 and ORF62 amplified in the present study were spliced using seqMan software in the DNAStar package, and the maximum likelihood tree was constructed using MEGA 7 with 1000 bootstrap replications. SNP analysis was also performed on 10 base loci of the ORF22 and ORF62 fragments in previous studies [28–30], which were used to identify the isolated VZV strain genotypes. The reference sequences for the phylogenetic analyses and SNP analyses, including Clades 1 to 5, were downloaded from the GenBank database (https://www.ncbi.nlm.nih.gov/). The Clade 1 reference strains included Dumas (X04370), Kel (DQ479954), MSP (AY548170), BC (AY548171), SD (DQ479953), 36 (DQ479958), 49 (DQ479959), 32P5 (DQ479961) and 32P22 (DQ479962); the Clade 2 reference strains included the wild-type strain pOka (AB097933), pOka-derived vaccine strain vOka (AB097932), VariVax (DQ008355) and Varilrix (DQ008354); the Clade 3 reference strains included 11 (DQ479955), 22 (DQ479956), and HJO (AJ871403); the Clade 4 reference strains included 8 (DQ479960) and DR (DQ452050); and the Clade 5 reference strains included CA123 (DQ457052).

For the sequences with mutations, reference sequences of ORF22 (MW545808) and ORF62 (AB097933) were downloaded from the GenBank database. Amino acids were translated and compared using DNAMAN 9 to explore whether these mutations lead to changes in amino acids.

### Statistical analysis

Statistical analysis was performed using the software Statistical Package for Social Sciences (SPSS, version 26; IBM Corporation, Armonk, USA). The normality of the quantitative data was tested by the Shapiro-Wilk test. Data with a normal distribution were statistically described by the mean ± standard deviation, and those without a normal distribution were statistically described by the median (interquartile spacing); qualitative data were described by a combination of numbers and percentages (%). Comparisons between two or more groups of rates were made using Fisher’s exact probability method, and a two-sided *P* value of less than 0.05 was considered statistically significant.

## GenBank accession numbers

The nucleotide sequences of all VZV isolates in this study have been submitted to GenBank with the accession numbers OR972777-OR972808 for ORF22 and OR972809-OR972840 for ORF62.

## Results

### Characteristics of the study population

A total of 39 patients with varicella were admitted to Ganzhou Fifth People’s Hospital from August 2021 to December 2022. The median age was 13.0 (8.0, 27.0) years, with a male-to-female ratio of 22:17. In the present study, patients with varicella were categorized into two groups, children and adults, according to their ages: 0–17 years and 18–55 years. There were 26 (66.7%) children and 13 (33.3%) adults.

There was a difference in the source of varicella patients between children and adults (*P* = 0.006), with 92.3% of adult VZV infections from inpatients (Table 2). In contrast, children were almost equally represented by outpatient and inpatient sources. The rate of discomfort, such as fever, sore throat, dizziness/headache, fatigue, or cough, was significantly greater in adults than in children (100.0% versus 57.7%, *P* = 0.007). Among them, the proportion of adults presenting with sore throat and fatigue was significantly greater than that of children (*P* < 0.05). Moreover, the incidence of complications after VZV infection was significantly greater in adults than in children (69.2% versus 7.7%, *P* < 0.001). There were three complications in children, including acute upper respiratory infection, acute tonsillitis, and skin infection, and nine complications in adults, including hepatitis, acute upper respiratory infection, pneumonia, thrombocytopenia, myocarditis, capillary leak syndrome, candida stomatitis, acute tonsillitis, and skin infection. Among these patients, adults were more likely to have hepatitis complications than children were (0.0% versus 46.2%, *P* = 0.001). There were no differences in the year of infection, sex distribution, or site of first varicella presentation between children and adults (*P* > 0.05).

**Table 2:** Characteristics of varicella patients in Ganzhou 2021–2022

Variable	Aged 0–17 years (%) *n* = 26	Aged 18–55 years (%) *n* = 13	*P* value
Years			
2021	15 (57.7)	3 (23.1)	0.051
2022	11 (42.3)	10 (76.9)	
Source of patients			
hospitalized patients	12 (46.2)	12 (92.3)	0.006^**^
outpatients	14 (53.8)	1 (7.7)	
Sex			
male	14 (53.8)	8 (61.5)	0.740
female	12 (46.2)	5 (38.5)	
The first site of chickenpox			
trunk	20 (76.9)	7 (53.8)	0.247
head	4 (15.4)	5 (38.5)	
limbs	2 (7.7)	1 (7.7)	
Presence of discomforts			
total	15 (57.7)	13 (100.0)	0.007^**^
fever	13 (50.0)	8 (61.5)	0.318
sore throat	3 (11.5)	6 (46.2)	0.039^*^
dizziness/headache	3 (11.5)	4 (30.8)	0.194
fatigue	2 (7.7)	5 (38.5)	0.030^*^
cough	1 (3.8)	3 (23.1)	0.099
Presence of complications			
total	2 (7.7)	9 (69.2)	< 0.001^***^
hepatitis	0 (0.0)	6 (46.2)	0.001^**^
acute upper respiratory infection	1 (3.8)	3 (23.1)	0.099
pneumonia	0 (0.0)	2 (15.4)	0.105
thrombocytopenia	0 (0.0)	2 (15.4)	0.105
myocarditis	0 (0.0)	1 (7.7)	0.333
capillary leak syndrome	0 (0.0)	1 (7.7)	0.333
candida stomatitis	0 (0.0)	1 (7.7)	0.333
acute tonsillitis	1 (3.8)	1 (7.7)	1.000
skin infection	1 (3.8)	1 (7.7)	1.000

^*^P value less than 0.05

^**^P value less than 0.01

^***^P value less than 0.001

Four (30.8%) adults with a mean age of 34.3 ± 5.9 years developed severe disease after VZV infection, and one died. All of them had concurrent hepatitis, and the results of relevant laboratory tests were well above the upper limit of the normal range: aspartate aminotransferase, 142.60 (2890.00) U/L; alanine aminotransferase, 835.73 ± 869.30 U/L; γ-glutamyl transferase, 160.15 ± 89.93 U/L; lactic dehydrogenase, 2077.50 ± 2018.58 U/L; and C-reactive protein, 33.10 ± 10.32 mg/L.

### Changes in laboratory tests in the study population

Nearly half (45.8%) of the children developed leukopenia after VZV infection. Accordingly, their median WBC count of 4.86 (3.71) × 10^9^/L was slightly below the lower limit of the normal reference range (Table S1). In contrast, adults had abnormal results for several laboratory tests: a mean eosinophil percentage of 0.25 (1.27)% and a mean Ca^2+^ of 2.16 ± 0.13 mmol/L were below the lower limit of the normal reference range, whereas a mean basophil percentage of 1.22 ± 0.63%, a median alanine aminotransferase of 65.40 (125.60) U/L, a median aspartate aminotransferase of 49.80 (54.50) U/L, a median γ-glutamyl transferase of 55.10 (121.15) U/L, a median lactic dehydrogenase of 308.80 (127.90) U/L, a mean C-reactive protein of 21.76 ± 16.55 mg/L and a mean glucose of 6.50 ± 1.73 mmol/L were above the upper limit of the normal reference range. These results were consistent with the laboratory test results, which revealed that 53.8% of adults with VZV infection had abnormal liver function and that 23.1% had hypocalcemia.

### Genotyping and phylogenetic analysis of Ganzhou strains

In this study, the ORF22 and ORF62 fragments of VZV were successfully amplified and sequenced in 32 plasma samples, with a success rate of 94.1% (32 out of 34). VZV genotyping was performed on the basis of the SNP sites in the amplified fragments (Table 3).

**Table 3:** Single nucleotide polymorphism (SNP) profiles of varicella virus were identified in this study

VZV strain	Clade	Position of ORF22					Position of ORF62				
		37,902	38,055	38,081	38,177	105,705	106,262	107,136	107,165	107,252	107,307
Dumas	1	A	T	A	G	T	T	T	C	T	T
pOka	2	G	C	C	A	T	T	T	T	T	C
HJO	3	A	T	A	G	T	T	T	T	T	T
DR	4	A	C	C	A	T	T	T	T	T	C
CA123	5	A	T	C	G	T	T	T	T	T	C
vOka^#^	2	G	C	C	A	C	C	C	T	C	C
Ganzhou isolates	2	G	C	C	A	T	T	T	T	T	C

^#^ vOka is a live attenuated vaccine derived from pOka. The nucleotide differences for ORF62 between pOka and vOka strains are clearly shown in the above table

Among them, four SNP sites were identified in the ORF22 fragment, which was consistent with those of pOka and vOka, indicating that these sequences belonged to Clade 2. In addition, six SNP sites were identified in the ORF62 fragment, which were consistent with those of pOka (Clade 2), CA123 (Clade 5), and DR (Clade 4). Taken together, these results indicate that these isolates belonged to Clade 2, as all of their SNP sites were consistent with those of pOka.

**Fig. 1:**
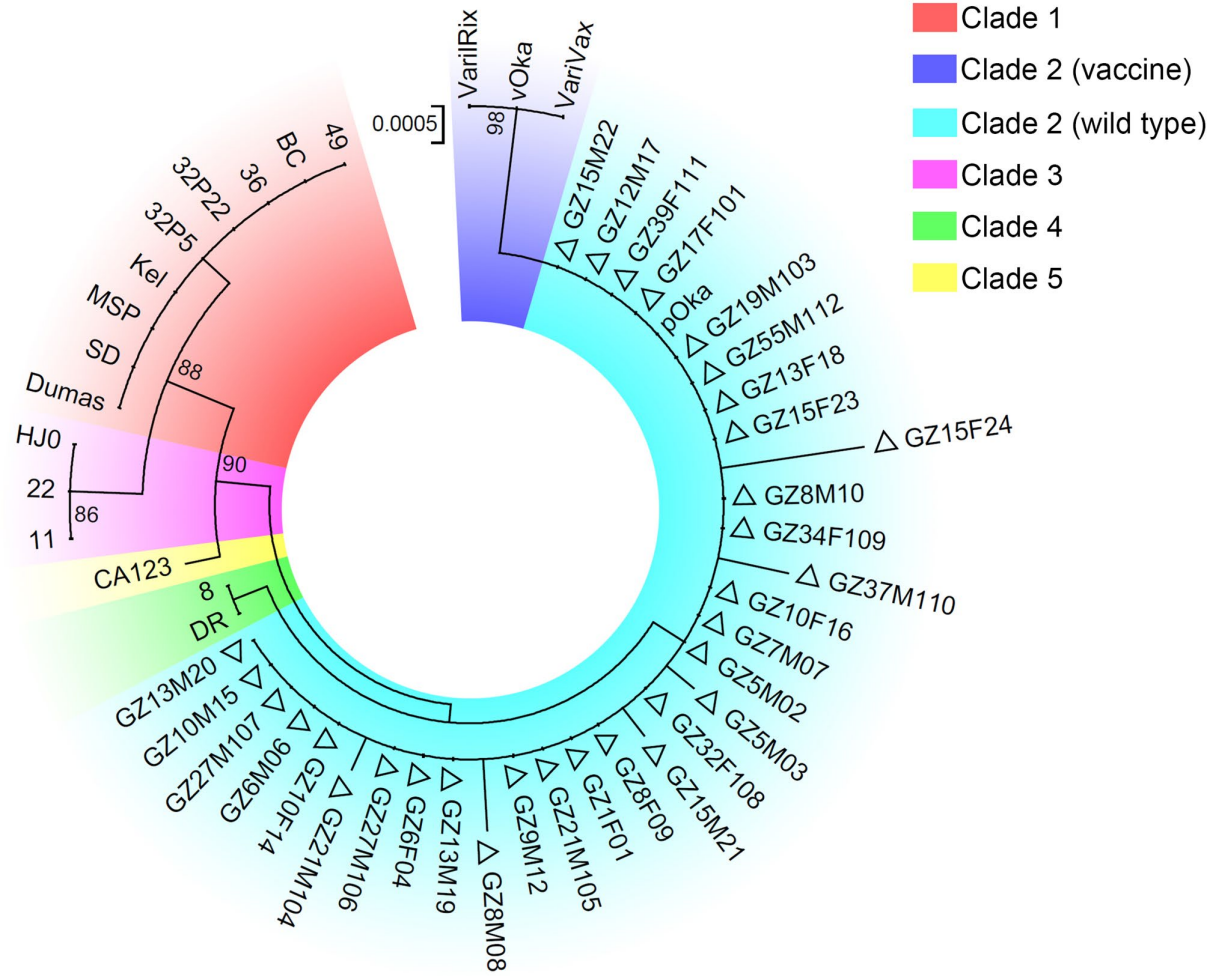
Maximum-likelihood tree based on the VZV ORF22 and ORF62 fragments. The triangles indicate the sequences amplified from varicella patients in Ganzhou, China. The bootstrap value of tree branches greater than 75% is shown at each node. The sectors with different colours indicate the different genotypes of VZV

A phylogenetic tree was further constructed by the maximum likelihood method to determine the genotypes and evolutionary relationships of the isolates (Fig. 1). Consistent with the results of the SNP locus analysis, the maximum likelihood tree revealed that the isolated virulent strains clustered with pOka but not with vOka, further confirming that all the clinical isolates in this study belonged to Clade 2.

### Mutational analysis of the Ganzhou strain

The maximum likelihood tree revealed that six VZV strains (GZ5M03, GZ8M08, GZ15M21, GZ15F24, GZ21M104, and GZ37M110) were genetically distant from the reference strain pOka and the other isolates, indicating that these isolates had mutations in their gene sequences (Fig. 1). Among these isolates, four (66.7%) were found in children, two (33.3%) were found in adults, and 83.3% of the variant patients were male. Further analysis revealed that the ORF22 and ORF62 fragments of the six mutant strains had a total of 12 base mutations, of which the ORF22 fragment contained 9 (75.0%) mutated bases and the ORF62 fragment contained 3 (25.0%) mutated bases (Table 4). Among the 12 mutated bases, the highest mutation prevalence from base A to base G (A→G) (41.7%), T→C incidence was the next highest (25.0%), followed by the incidence of mutations in G→A, C→Y, A→T and T→A (8.3% each). Most (50.0%) mutants had 2-base mutations, whereas the GZ15F24 mutant had 4-base mutations.

**Table 4:** Genetic diversity analysis of VZV mutants in Ganzhou, China

Isolates	Fragments^*^	Nucleotide positions	Amino acid positions	Base mutations	Results^#^
GZ5M03	ORF22	37,925	1281	A→G	SN
GZ8M08	ORF22	37,940	1286	G→A	Met→Ile
GZ8M08	ORF22	37,990	1303	A→T	Gln→Leu
GZ15F24	ORF22	37,891	1270	A→G	Glu→Gly
GZ15F24	ORF22	37,939	1286	T→C	Met→Thr
GZ15F24	ORF62	106,086	1016	A→G	SN
GZ15F24	ORF62	107,070	688	T→C	SN
GZ15M21	ORF62	107,122	671	A→G	Val→Ala
GZ21M104	ORF22	37,986	1302	C→Y	Arg→Arg/Cys
GZ21M104	ORF22	38,143	1354	T→A	Val→Glu
GZ37M110	ORF22	37,974	1298	T→C	Tyr→His
GZ37M110	ORF22	38,133	1351	A→G	Thr→Ala

^*^ According to the reference sequences of ORF22 (MW545808) and ORF62 (AB097933)

^#^ Ala, alanine; Arg, arginine; Cys, cysteine; Gln, glutamine; Glu, glutamic acid; Gly, glycine; His, histidine; Ile, isoleucine; Leu, leucine; Met, methionine; SN, synonymous mutation; Thr, threonine; Tyr, tyrosine; Val, valine

Among the mutated sequences, 75.0% were missense mutations, and 25.0% were synonymous mutations (Table 4). Eight of the nine bases mutated in the ORF22 fragment were missense mutations (88.9%), and one of the three bases mutated in the ORF62 fragment (33.3%) was a missense mutation. In the isolates from children, base mutations at sites 37,940 and 37,990 in GZ8M08 resulted in changes in amino acids 1286 and 1303 encoded by the ORF22 fragment, with the former changing from methionine to isoleucine and the latter changing from glutamine to leucine; base mutations at sites 37,891 and 37,939 in GZ15F24 resulted in changes in amino acids 1270 and 1286, with the former changing from glutamic acid to glycine and the latter changing from methionine to threonine; a base mutation at site 107,122 of isolate GZ15M21 resulted in a change in amino acid 671, which is encoded by the ORF62 fragment from valine to alanine. In the isolates from adults, the mutations at sites 37,986 and 38,143 of GZ21M104 resulted in changes in amino acids 1302 and 1354 encoded by the ORF22 fragment, the former from arginine to arginine/cysteine and the latter from valine to glutamic acid; base mutations at positions 37,974 and 38,133 in isolate GZ37M110 resulted in changes in amino acids 1298 and 1351 of the ORF22 fragment, the former from tyrosine to histidine and the latter from threonine to alanine.

## Discussion

VZV was highly contagious in individuals without a history of varicella and could be reactivated in human ganglion cells after initial infection [31]. Primary VZV infection resulting in varicella was usually considered a mild childhood disease; however, data from past studies had shown that varicella in adults was 25 times more likely to be severe (and possibly even fatal) than those in children [7, 32]. The present study revealed that varicella in children often presented as a self-limiting disease, with the main symptom being fever. However, four adults developed severe diseases, one of whom died. These results were consistent with those of previous studies. Moreover, the present study also compared the results of laboratory markers between children and adults and revealed that test results regarding liver function, such as alanine aminotransferase, aspartate aminotransferase, γ-glutamyl transferase, and lactic dehydrogenase, were higher than the upper limit of the normal reference range; however, this phenomenon was not found among children. Therefore, more attention should be given to adult varicella patients because they might be at greater risk of developing serious illnesses, especially liver dysfunction.

Molecular epidemiological information on VZV was crucial for characterizing endemic strains, tracing transmission routes, distinguishing genotypes, and exploring evolutionary patterns [7, 22, 33]. Previous studies have shown that Clade 2 was predominant in China, and the live attenuated vaccine used to prevent varicella in China was designed on the basis of the Oka strain of Clade 2 [18, 34, 35]. In a small number of vaccinated children, the varicella vaccine virus reactivates and causes herpes zoster, sometimes with meningitis or meningoencephalitis. Occasionally the herpes zoster rash can be extensive enough to be confused with an early varicella rash. Cases have been reported in the United States, Germany, Greece, Switzerland and Japan [36]. In the present study, all clinical VZV isolates belonged to the highly homogeneous wild-type Clade 2, and no vaccine strain infections were detected. This indicates that the current varicella vaccine in Ganzhou should continue to be promoted to reduce the incidence of varicella, as it remains effective. Moreover, the genomic sequencing technology developed in Ganzhou and described in this report will enable physicians to diagnose any vaccine-related adverse events that occur in Chinese children.

Several studies have shown that the epidemiological subtypes of VZV in peripheral regions of China, such as Xinjiang, Xizang and Guangdong Provinces, were more complex than those in internal regions [23, 34, 37, 38]. Three clades, including Clade 2, Clade 4 and Clade 5, were found to be prevalent in Guangdong Province [34]. Ganzhou, the sample site of the present study, borders Guangdong Province, with a border of 1400 km. Although only Clade 2 had been found to be prevalent in Ganzhou, continuous monitoring of the molecular characteristics of VZV was urgently needed to avoid the complication of the VZV genotype, as cross-border activities were quite common in this region.

It was hypothesized that VZV appeared in early primitive humans in Africa approximately 7 million years ago and evolved with primates [24]. The VZV genome was considered highly conserved, but it was still evolving, with mutations occurring occasionally, some of which were fixed and some of which were random [26]. In this study, twelve previously unidentified mutated bases were present in the ORF22 and ORF62 fragments of the six isolates, with the ORF22 fragment having a more significant percentage of mutated bases than the ORF62 fragment (75.0% versus 25.0%). Moreover, the incidence of amino acid changes resulting from mutated bases was greater in the ORF22 fragment than in the ORF62 fragment (88.9% versus 33.3%). Overall, the frequency of prevalent genetic variation in VZV in the population is low, possibly due to its low reproduction rate in infected hosts, thus limiting the frequency of introducing new mutations.

Two limitations should be considered when interpreting the results of the present study. First, there were no tests to determine whether the differences in laboratory test results between children and adults were statistically significant due to the small sample sizes of these subgroups. Second, the ORF22 and ORF62 fragments of VZV among the two patients were failed to be amplified, probably due to the low viral load in the plasma samples. In future studies, the simultaneous use of vesicular fluid and blood samples may enhance the amplification efficiency of VZV DNA.

## Conclusions

In this study, clinical symptoms, disease progression, and laboratory test results of varicella patients were retrospectively analyzed to explore the differences in disease between children and adults, and molecular epidemiology was used to characterize the genetic variation of VZV isolates in Ganzhou. All isolates were Clade 2 wildtype with high homogeneity. Adults were more severely infected with VZV than children, with a greater tendency to involve the liver and a higher risk of developing severe disease. These results reveal that preventive and therapeutic measures should be intensified for adult VZV-susceptible populations and those at high risk of severe disease after infection.

## Electronic supplementary material

Below is the link to the electronic supplementary material.

Supplementary Material 1

## Data Availability

No datasets were generated or analysed during the current study.

## Abbreviations

ORF: Open reading frame
SNP: Single-nucleotide polymorphism
VZV: Varicella-zoster virus
