# Supplementary material for: Clinical symptoms and molecular epidemiologic characteristics of varicella patients among children and adults in Ganzhou, China
Source: Virol J. 2025 Feb 21;22:44. doi: 10.1186/s12985-025-02661-6 (PMC11844084; doi:10.1186/s12985-025-02661-6)
Supplement: Supplementary file 1 — Supplementary Material 1 [file 12985_2025_2661_MOESM1_ESM.docx]

**Table S1. The laboratory test results of varicella patients in Ganzhou, China, 2021-2022.**

| Laboratory tests | Normal reference range | Test results | | |
| --- | --- | --- | --- | --- |
|  |  | Aged 0-17 years | Aged 18-55 years | |
| White blood cell count ^*^, ×10^9^/L | 5-12; 3.5-9.5 | 4.86 (3.71) ↓ | 5.67 (3.12) |  |
| Neutrophil percentage, % | 50-70 | 51.80±13.50 | 56.36±15.52 |  |
| Lymphocyte percentage, % | 20-40 | 34.76±13.14 | 33.42±17.25 |  |
| Monocyte percentage, % | 3-12 | 11.23±3.28 | 7.64±4.17 |  |
| Eosinophil percentage, % | 0.5-5.0 | 0.70 (2.17) | 0.25 (1.27) | ↓ |
| Basophil percentage, % | 0-1 | 0.65 (0.60) | 1.22±0.63 | ↑ |
| Neutrophil count, ×10^9^/L | 2-7 | 2.43 (2.70) | 3.30 (1.85) |  |
| Lymphocyte count, ×10^9^/L | 0.8-4 | 1.84 (1.00) | 1.65 (2.54) |  |
| Monocyte count, ×10^9^/L | 0.12-1.2 | 0.53 (0.28) | 0.50±0.30 |  |
| Eosinophil count, ×10^9^/L | 0.02-0.50 | 0.03 (0.11) | 0.02 (0.06) |  |
| Basophil count, ×10^9^/L | 0-0.1 | 0.04 (0.02) | 0.07 (0.08) |  |
| Red blood cell count, ×10^12^/L | 3.5-5.0 | 4.68±0.52 | 4.61±0.63 |  |
| Hemoglobin ^* #^, g/L | 151-163; 124-165 | 133.96±11.42 | 133.58±14.27 |  |
| Hematocrit, % | 37-54 | 38.45±3.14 | 38.32±3.50 |  |
| Mean corpuscular volume, fL | 80-100 | 85.20 (8.45) | 87.35 (2.33) |  |
| Mean corpuscular hemoglobin, pg | 27-34 | 29.65 (3.10) | 30.20 (2.80) |  |
| MCHC, g/L | 320-360 | 348.33±8.45 | 345.00 (17.25) |  |
| RDW-CV, % | 11-16 | 13.06±1.21 | 13.25±1.24 |  |
| RDW-SD, fL | 35-56 | 37.60 (3.22) | 39.02±4.25 |  |
| Platelet count, ×10^9^/L | 125-350 | 180.50 (108.50) | 144.08±64.64 |  |
| Mean platelet volume, fL | 6.5-12 | 9.24±1.00 | 9.75±0.60 |  |
| Platelet distribution width, fL | 9-17 | 15.76±0.43 | 15.90±0.54 |  |
| Procalcitonin, % | 0.108-0.282 | 0.19±0.06 | 0.14±0.07 |  |
| Immature granulocyte count, ×10^9^/L | 0-999.99 | 0.00 (0.01) | 0.01 (0.01) |  |
| Immature granulocyte percentage, % | 0-100 | 0.10 (0.18) | 0.10 (0.18) |  |
| Platelet-large cell count, ×10^9^/L | 30-90 | 42.63±15.25 | 37.00±18.09 |  |
| Platelet-large cell percentage, % | 11-45 | 21.46±6.91 | 26.57±5.31 |  |
| Total bilirubin, μmol/L | 3.42-20.5 | 6.25 (4.10) | 7.80 (6.50) |  |
| Direct bilirubin, μmol/L | 0-6.84 | 1.79±1.00 | 2.10 (1.90) |  |
| Indirect bilirubin, μmol/L | 1.7-13.2 | 4.90 (2.50) | 6.02±2.25 |  |
| Alanine aminotransferase, U/L | 7-40 | 16.65 (8.33) | 65.40 (125.60) | ↑ |
| Aspartate aminotransferase, U/L | 13-35 | 21.45 (11.90) | 49.80 (54.50) | ↑ |
| Total protein, g/L | 65-85 | 72.48±6.16 | 66.47±8.22 |  |
| Albumin, g/L | 38-54 | 45.14±3.12 | 41.36±5.22 |  |
| Globulin, g/L | 20-30 | 26.54±5.66 | 25.12±5.48 |  |
| Alkaline phosphatase ^*^, U/L | 40-610; 40-150 | 199.00±88.52 | 71.80 (40.90) |  |
| γ-glutamyl transferase, U/L | 7-45 | 14.11±3.45 | 55.10 (121.15) | ↑ |
| Cholinesterase, U/L | 3600-12000 | 8247.93±1883.76 | 8419.15±2572.89 |  |
| Lactic dehydrogenase ^*^, U/L | 120-250; 103-227 | 242.09±62.10 | 308.80 (127.90) | ↑ |
| Creatine kinase, U/L | 24-194 | 78.55 (35.12) | 103.90 (229.05) |  |
| Creatine kinase isoenzymes, U/L | 0-25 | 18.38±7.44 | 20.10 (48.65) |  |
| Plasma urea, mmol/L | 1.43-7.14 | 4.05±1.28 | 3.98±1.35 |  |
| Plasma uric acid, μmol/L | 155-357 | 344.81±75.59 | 298.90 (76.75) |  |
| Plasma creatinine, μmol/L | 35-80 | 60.59±19.39 | 78.06±16.81 |  |
| C-reactive protein, mg/L | 0-8.2 | 6.60 (9.71) | 21.76±16.55 | ↑ |
| Glucose, mmol/L | 3.89-6.11 | 5.79±1.10 | 6.50±1.73 | ↑ |
| K^+^, mmol/L | 3.5-5.5 | 4.24±0.45 | 3.69±0.50 |  |
| Na^+^, mmol/L | 135-145 | 140.82±1.59 | 137.18±3.64 |  |
| Cl^-^, mmol/L | 95-105 | 100.33 (2.63) | 97.73±3.95 |  |
| Ca^2+^, mmol/L | 2.2-2.75 | 2.30±0.17 | 2.16±0.13 | ↓ |

↑ Above the normal reference range.

↓ Below the normal reference range.

^*^ The reference ranges of testing items for patients aged 0-17 years were different from those aged 18-55. The former was the reference range of testing items for patients aged 0-17 years, and the latter was for patients aged 18-55 years.

^#^ The normal reference range of hemoglobin was calculated based on the number of females and males, because the normal reference range of hemoglobin of females was 115–150 g/L, and the normal reference range of hemoglobin of males was 130–175 g/L.

MCHC, mean corpuscular hemoglobin concentration; RDW-CV, red blood cell distribution width-coefficient of variation; RDW-SD, red blood cell distribution width-standard deviation.
